# Standardization of Care is Key: Reducing CLABSI in Pediatric Patients

**DOI:** 10.1097/pq9.0000000000000498

**Published:** 2021-09-02

**Authors:** Jennifer D. Thomas, Stacy L. Flanders

**Affiliations:** From the Norton Children’s Hospital, Louisville, Ky.

## Abstract

Children’s Hospitals’ Solutions for Patient Safety (SPS) is a network of over 140 children’s hospitals who share the vision of working together to eliminate serious harm across all pediatric hospitals. The SPS network is built on the fundamental belief that by sharing successes and failures transparently and learning from one another, children’s hospitals can achieve their goals more effectively and quickly than working alone. Each year, SPS hosts National Learning Sessions to which members are invited to submit abstracts describing relevant safety research or improvement work. The following abstracts were among the top submitted for the SPS Spring 2021 National Learning Session.

## Background:

Central line-associated bloodstream infections (CLABSIs) cause increased antibiotic usage, hospital length of stay, hospital costs, and, can contribute to patient deaths. The average cost of a CLABSI is around $55,000. Over the last 2 years, Norton Children’s Hospital (NCH) has averaged 19 CLABSIs per year, (average rate of 1.1 per 1000 central line days). Mucosal Barrier Injury CLABSIs have accounted for 50% of our CLABSIs.

## Objectives:

Our aim was to reduce CLABSI by standardizing the team performing central line audits and dressing changes, and to implement an oral care bundle for pediatric oncology patients.

## Methods:

The Vascular Access Team began performing central venous line (CVL) dressing changes and daily audits of all CVL's using the Kamishibi process. Noncompliance is communicated to unit leaders, who are then responsible for assessing the five “whys” to determine the root cause. Standardization has resulted in analysis of the most common reasons for noncompliance and allowed the team to work together to improve processes and to remove barriers. To address Mucosal Barrier Injury CLABSIs, an oral care bundle was developed for pediatric oncology patients. Oral care boxes containing kid-friendly supplies were obtained and provided to patients upon admission, which has resulted in an increase in oral care compliance.

## Results:

Standardization is key. Utilization of a core group of staff to perform daily audits of CVL maintenance and dressing changes, along with implementation of a standardized evidence-based oral care bundle, has resulted in a 60% reduction in CLABSI in the 6 months post-implementation.

**Fig. 1.**
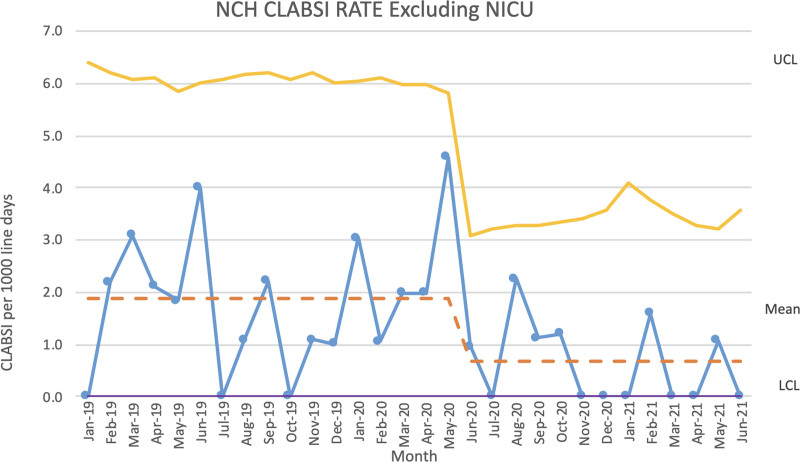
Norton Children’s Hospital CLABSI Rate Excluding NICU.

